# Heterozygous p.Asp50Asn mutation in the *GJB2* gene in two Cameroonian patients with keratitis-ichthyosis-deafness (KID) syndrome

**DOI:** 10.1186/1471-2350-14-81

**Published:** 2013-08-07

**Authors:** Ambroise Wonkam, Jean Jacques N Noubiap, Jason Bosch, Collet Dandara, Geneviève Bengono Toure

**Affiliations:** 1 Division of Human Genetics, Department of Clinical Laboratory Sciences, Faculty of Health Sciences, University of Cape Town, Cape Town, South Africa; 2 Faculty of Medicine and Biomedical Sciences, University of Yaoundé I, Yaoundé, Cameroon; 3 Division of Human Genetics, Faculty of Health Sciences, University of Cape Town, Anzio Road, Observatory 7925, Cape Town, South Africa

**Keywords:** KID syndrome, *GJB2* gene, p.Asp50Asn mutation, Africa, Cameroon

## Abstract

**Background:**

Keratitis-Ichthyosis-Deafness (KID) syndrome (OMIM 148210) is a congenital ectodermal defect that consists of an atypical ichthyosiform erythroderma associated with congenital sensorineural deafness. KID appears to be genetically heterogeneous and most cases are caused by *GJB2* mutations. Mutations in African patients have been rarely described.

**Case presentation:**

We report on two unrelated Cameroonian individuals affected with sporadic KID, presenting with the classic phenotypic triad. The two patients were heterozygous for the most frequent p.Asp50Asn mutation. This first report in patients from sub-Saharan African origin supports the hypothesis that the occurrence of KID due to p.Asp50Asn mutation in *GJB2* seems not to be population specific.

**Conclusions:**

Our finding has implication in medical genetic practice, specifically in the molecular diagnosis of KID in Africans. These cases also reveal and emphasize the urgent need to develop appropriate policies to care for patients with rare/orphan diseases in Sub-Saharan Africa, as many of these cases become more and more recognizable.

## Background

The Keratitis-ichthyosis-deafness (KID) syndrome (OMIM 148210 and 242150) is a rare congenital ectodermal disorder of unknown prevalence. Approximately 100 cases have been reported in the world to date [1]. KID appears to be genetically heterogeneous and most cases are caused by mutations in the connexin 26 gene, *GJB2*. Connexins are membrane proteins with a common structure consisting of four transmembrane domains linked by one cytoplasmic and two extracellular loops (namely, EC1, and EC2, respectively), with both cytoplasmic N-terminal and C-terminal [2]. Different mutations in the genes encoding connexins can disturb the gap junction system of one or several ectodermal epithelia, which in the case in KID syndrome, where the epidermis, the inner ear and the corneal epithelium are affected [3].

Skinner et al. reviewed 18 affected patients and proposed the acronym ‘KID syndrome’ to describe three main symptoms, ichthyosis, vascularizing keratitis, and often profound sensorineural hearing loss [4]. KID is genetically heterogeneous and is caused by missense mutations in the connexin (CX) genes *GJB2* and *GJB6*, which cluster at chromosome 13q11-q12 and encode the closely related gap junction β-2 protein (coded by CX26) and β-6 protein (coded by CX30) [5,6]. Inheritance of KID syndrome is usually sporadic but autosomal recessive and dominant cases have been reported [5].

Mutations in cases of KID syndrome have been rarely reported in patients of African descent [7], but p.Asp50Asn mutation in the *GJB2* gene have been previously only reported in a black patient from the Emirates [8]. In this article, we report on two unrelated Cameroonian patients with KID syndrome presenting with heterozygous p.Asp50Asn mutation in the *GJB2* gene.

## Case presentation

### Patients and methods

We previously published on aetiological factors of congenital hearing loss on 582 Cameroonians [9]. Amongst them, two young patients presented with clinical features of KID syndrome.

### DNA amplification and mutation analysis

Genomic DNA samples were extracted from peripheral blood of the two patients, using Puregene blood Kit® (Qiagen, USA), following the manufacturer’s protocol and this was carried out in the Molecular Diagnosis Laboratory of the Gyneco-Obstetric and Paediatric Hospital of Yaoundé, Cameroon. The *GJB2* gene was amplified following the method of Liu et al. [10]. Exon 2 was amplified and then sequenced using an ABI 3130XL Genetic Analyze® automated sequencer (Applied Biosystems, USA), in the Molecular Research laboratory in the Division of Human Genetics, University of Cape Town, South Africa.

### Clinical data

#### Patient 1

**Figure 1:**
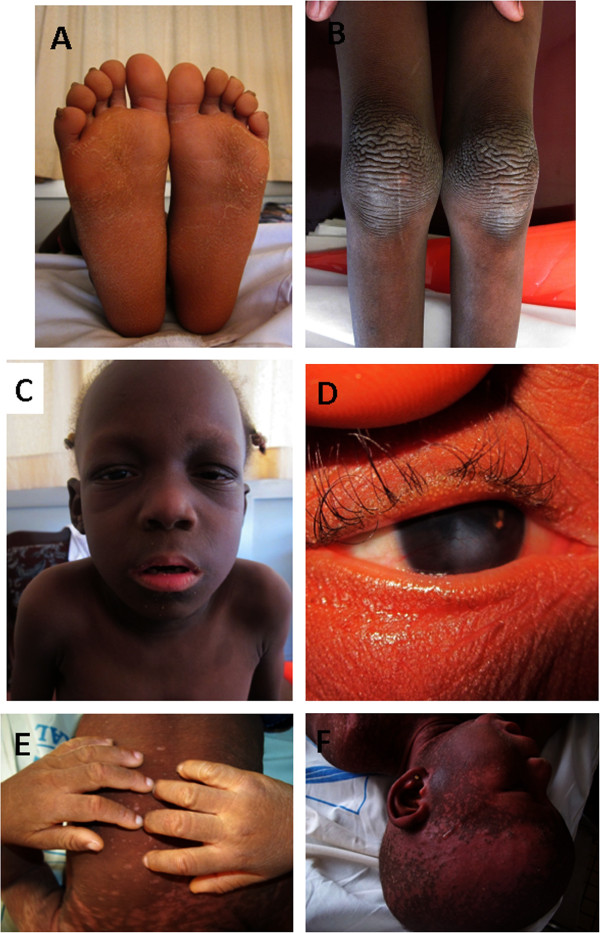
**Illustrations of some clinical features of the two Cameroonian KID cases (Case 1; panels A-D; Case 2 panels E and F). A)** Keratoderma of the soles **B)** Rippled hyperkeratotic plaques on the knees; **C)** Hypotrichosis of the eyelashes and eyebrows; **D)** Mild vascularizing keratitis; **E)** Hyperkeratosis of the hands; **F)** Alopecia, hypotrichosis, ichthyosiform erythrokeratoderma.

A five-year-old girl that presented with a profound bilateral sensorineural deafness diagnosed at 2 years old. She was born at term to unrelated healthy parents after an uneventful pregnancy and normal vaginal delivery, and presented at birth with generalized erythema. She had a history of chronic otitis externa and hypohidrosis. Her psychomotor development was normal; however, physical examination revealed a generalized thickened skin and xeroderma, palmoplantar keratoderma and rippled hyperkeratotic plaques on the knees and elbows (Figure 1A-D). She had aged facial appearance, hypotrichosis (sparse of eyelashes and eyebrows), and hyperkeratosis lesions in the external auditory canal. Ophthalmologic examination revealed a mild vascularizing keratitis which explained her photophobia and reduced visual acuity. Oral examination showed dental dysplasia and histopathological examination of the skin revealed an acanthotic dyskeratosis. The parents were non-consanguineous and there was no family history of similar condition.

#### Patient 2

A two-year-old girl that presented with a prelingual bilateral profound sensorineural deafness. She was born at term after an uncomplicated pregnancy and delivery. Since two months of age, she presented with thick, reddened patches of the skin that were dry and scaly. The thickness of the skin gradually increased as she grew older. At the time of presentation at the health facility physical examination revealed generalized ichthyosiform erythrokeratoderma, palmoplantar keratoderma, alopecia universalis and atrichosis (absence of eyelashes and eyebrows) (Figure 1E-F). Joint mobility of the elbows, knees and ankles was seriously reduced by keratoderma. She had photophobia and ocular irritation, and an ophthalmologic examination revealed a vascularizing keratitis. The intraoral examination was normal. She had no major neurological abnormalities and her non-consanguineous parents and young sister were all healthy and there was no family history of similar clinical presentation.

On familial history, the two patients were unrelated: their parents originated from, and live in, two geographically distinct of area of Cameroon (Western and Centre provinces, about 400 km apart). The parents belonged to two different ethno-linguistic groups: Bantu and Nilo-Sahelian, respectively.

### Genetic analysis

**Figure 2:**
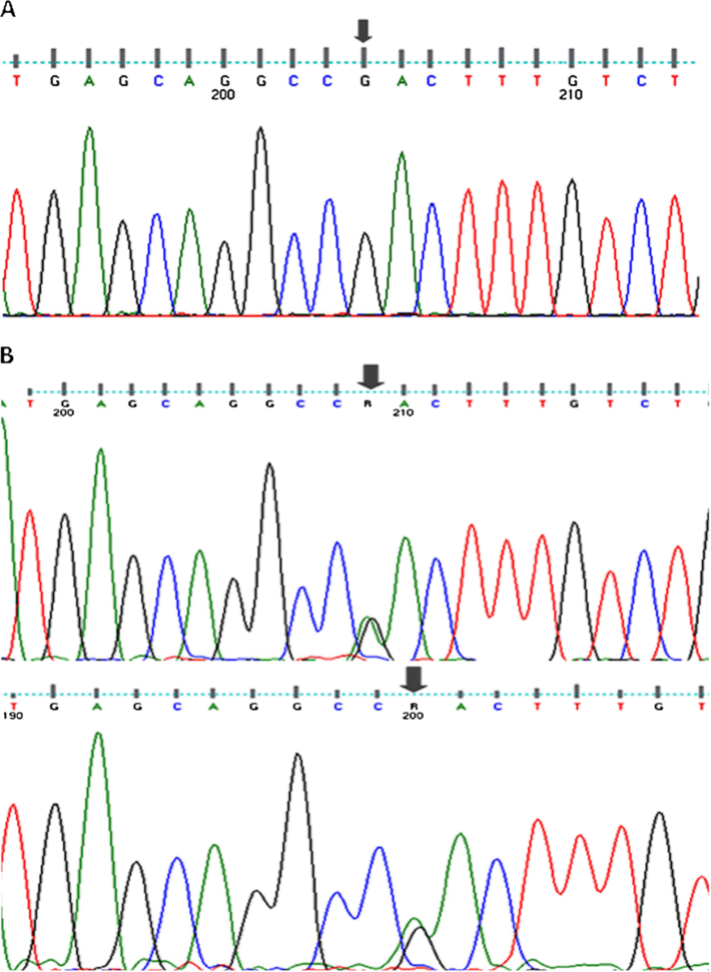
**Mutation analysis of *GJB2 *in the two Cameroonian individuals affected with sporadic KID.** Panel **A**: Sequence chromatograms of *GJB2* from unaffected individual; Panel **B**: Sequence chromatograms from affected patients depicting the heterozygous transition 148G → A at codon 50 encoding asparagine instead of aspartic acid (p.Asp50Asn) (Panel **B**).

Analysis of *GJB2* exon 2 in the genomic DNA of the two unrelated patients revealed a heterozygous missense mutation c.148G > A, resulting in a putative amino acid change from aspartic acid (GAC) to asparagine (AAC) in codon 50 p.Asp50Asn (Figure 2). p.Asp50Asn were not present in more than 180 unrelated individuals who were screened for recessive deafness mutations or in 60 healthy control persons of Cameroonian origin [*unpublished results*].

Moreover, sequences data from both patients did not show any other common changes, but the p.Asp50Asn variant. The DNA sequence in patient 1 did not have any other changes than the p.Asp50Asn variant, while patient 2 was also heterozygote for the most common non pathogenic polymorphism, g.3318-34C > T. Thus, there was no further need to compare the haplotypes from the two patients to establish unrelatedness.

### Discussion

To the best of our knowledge, this is the first report of sub-Saharan African patients with KID syndrome due to the p.Asp50Asn mutation in *GJB2.*

*GJB2* mutations responsible for non-syndromic hearing loss have been reported in many parts of the world with marked variations in distribution patterns among different ethnic groups, with a propensity to occur frequently in some population groups (in Europe, North America and Asia) [10,11], while seemingly absent in African populations [12]. Contrary to *GJB2* mutations causing autosomal recessive non-syndromic hearing loss (HL) which are ethnically specific because of founder effect in some specific populations, *GJB2* mutations responsible for syndromic hearing loss seem not to be population specific [5].

The two patients reported here presented with the classic phenotypic triad of KID syndrome including diffuse hyperkeratotic erythroderma, vascularizing keratitis, and profound bilateral sensorineural hearing loss. Several other features were associated including alopecia, hypotrichosis and hypohydrosis, porokeratotic eccrine ostial and dermal duct nevus, follicular occlusion triad and dental anomalies previously [13,14]. Moreover, the p.Asp50Asn mutation has also been reported in KID patients with Dandy-Walker malformation [15]; although brain imaging was not performed, the two cases described here did not have neurologic features that would suggest the occurrence of Dandy-Walker malformation. The p.Asp50Asn mutation has been reported mostly in sporadic cases, but also in a case of autosomal dominant inheritance [5]. In a cohort of 14 patients affected with KID syndrome originating from 11 families, where parent to child transmission in families was verified by molecular analysis, twelve patients (86%) were heterozygous for the p.Asp50Asn mutation. The disease was sporadic in 64%, whereas 36% was familial, suggestive of autosomal dominant inheritance with one parent clinically affected in all the families. A family with p.Asp50Asn mutation was suggestive of germinal mosaicism, as the parent was clinically normal [16]. Similar report of germinal mosaicism was also reported in family with dizygotic twins suffering from a lethal form of KID; the two patients were heterozygous for the p.G45E mutation of *GJB2*, whereas the mutation was not detected in the two parents [7]. The two pairs of Cameroonian parents did not consent for their molecular analysis, claiming in both cases, that this will not have any implication in improving the care of their KID-affected children. However, since none of the parents were clinically affected, and in absence of reported proven familial case with reduce penetrance, mutations in these two Cameroonian cases are most likely *de novo*.

p.Asp50Asn appears to be the most prevalent mutation in unrelated KID patients of Caucasian ancestry [5,16], and the two unrelated Cameroonian cases provide additional information that this mutation may also be important in African populations. The amino acid replacement, in p.Asp50Asn mutation, occurs in the highly conserved first extracellular loop of CX26, which is crucial for voltage gating and connexon-connexon interactions [2,17]. The majority of dominantly-acting connexin mutations, associated with autosomal dominant syndromic HL are situated in this domain and are missense mutations, while the majority that cause recessive HL are nonsense mutations or small deletions [17]. Moreover, it has been observed that alteration of calcium ion fluxes due to the effects of mutations such as p.Asp50Asn, result in cell death by necrosis [18]. In addition, other functional analyses showed that p.Asp50Asn have consequences for protein localization and gap junction permeability [19]. However, more evidences are needed to associate the variable phenotypes observed in KID with effects on protein trafficking or gap junction permeability.

The management of KID syndrome in Cameroon as in other low-income countries is a critical issue. The most beneficial treatment for the profound hearing impairment in our patients could be cochlear implantation [20], a procedure that is unavailable in Cameroon. There is no universal medical insurance in Cameroon and even hearing aids are not affordable for most patients. Keratitis (also observed in the two cases) can result in progressive decline of visual acuity and may eventually lead to blindness which combined with a profound hearing loss constitute a disastrous disability. In addition, a life-long follow-up of these patients is necessary because the KID syndrome is associated with malignant tumours, particularly squamous cell carcinoma [1]. Although the provision of service for medical care for KID patients is limited in Cameroon, better awareness of the disease within the region would help patients if upon diagnosis, better prevention of the worst effects of disease can be instituted, which besides follow-up for cancerous lesions, would include simple preventative measures against fatal septicemia from recurrent skin infections; specifically for the keratitis; there is a need to develop an appropriate specialist services in the country that could help to prevent blindness.

## Conclusion

In conclusion, our report supports the occurrence of KID due to p.Asp50Asn mutations in *GJB2* in Africans and seems to indicate that this mutation is not ethically specific. These cases reveal and emphasize the urgent need to develop appropriate policies to care for patients with rare/orphan diseases in Sub-Saharan Africa, as many of these cases become more and more recognizable.

## Consent

The study was performed in accordance with the guidelines of the Helsinki Declaration and was approved by the National Ethics Committee of Cameroon (ethics approval N°123/CNE/SE/2010), and the Faculty of Health Sciences Research Ethics Committee, University of Cape Town, HREC REF: 080/2011. Written informed consents were obtained from patient parents for publication and the accompanying images. Copies of the written consents are available for review.

## Abbreviations

KID: Keratitis-ichthyosis-deafness; EC1 and EC2: Extracellular loops 1 and 2; CX: Connexion.

## Competing interests

The authors declaim no competing interests.

## Authors’ contributions

AW and GBT designed the study, raised fund, assured general supervision of the research group, drafted the manuscript and compelled the revisions; JJNN acquired clinical data and performed DNA extraction and draft the manuscript, JB and CD performed and supervised the molecular analysis of *GJB2* gene. All the authors revised and approved the final version the manuscript.

## Pre-publication history

The pre-publication history for this paper can be accessed here:

http://www.biomedcentral.com/1471-2350/14/81/prepub
